# Outcomes of STEMI patients with chronic kidney disease treated with percutaneous coronary intervention: the Malaysian National Cardiovascular Disease Database – Percutaneous Coronary Intervention (NCVD-PCI) registry data from 2007 to 2014

**DOI:** 10.1186/s12872-018-0919-9

**Published:** 2018-09-24

**Authors:** Muhammad Dzafir Ismail, Maisarah Jalalonmuhali, Zaid Azhari, Jeevitha Mariapun, Zhen-Vin Lee, Imran Zainal Abidin, Wan Azman Wan Ahmad, Ahmad Syadi Mahmood Zuhdi, On behalf of NCVD-PCI investigators

**Affiliations:** 1 Division of Cardiology, Department of Medicine, University Malaya Medical Centre, 59100 Kuala Lumpur, Malaysia; 2 Division of Nephrology, Department of Medicine, University Malaya Medical Centre, 59100 Kuala Lumpur, Malaysia; 3 Department of Social and Preventive Medicine, University of Malaya, 50603 Kuala Lumpur, Malaysia

## Abstract

**Background:**

Patients with renal impairment often left out from most major clinical trials assessing the optimal treatment for ST-elevation myocardial infarction (STEMI). Large body of evidence from various cardiovascular registries reflecting more ‘real-world’ experience might contribute to the knowledge on how best to treat this special cohort. We aim to analyze the outcomes of Malaysian STEMI patients with renal impairment treated with coronary angioplasty.

**Methods:**

Utilizing the Malaysian National Cardiovascular Disease Database-Percutaneous Coronary Intervention (NCVD-PCI) registry data from 2007 to 2014, STEMI patients treated with percutaneous coronary intervention (PCI) were stratified into presence (GFR < 60 mls/min/1.73m^2^) or absence (GFR ≥ 60 mls/min/1.73m^2^) of chronic kidney disease (CKD). Patient’s demographics, extent of coronary artery disease, procedural data, discharge medications, short (in-hospital) and long (1 year) term outcomes were critically assessed.

**Results:**

A total of 6563 patients were included in the final analysis. STEMI CKD cohort was predominantly male (80%) with mean age of 61.02 ± 9.95 years. They had higher cardiovascular risk factors namely diabetes mellitus (54.6%), hypertension (79.2%) and dyslipidemia (68.8%) in contrast to those without CKD. There were notably higher percentage of CKD patients presented with Killip class 3 and 4; 24.9 vs 8.7%. Thrombolytic therapy remained the most commonly instituted treatment regardless the status of kidney function. Furthermore, our STEMI CKD cohort also was more likely to receive less of evidence-based treatment upon discharge. In terms of outcomes, patients with CKD were more likely to develop in-hospital death (OR: 4.55, 95% CI 3.11–6.65), MACE (OR: 3.42, 95% CI 2.39–4.90) and vascular complications (OR: 1.88, 95% CI 0.95–3.7) compared to the non-CKD patients. The risk of death at 1-year post PCI in STEMI CKD patients was also reported to be high (HR: 3.79, 95% CI 2.84–5.07).

**Conclusion:**

STEMI and CKD is a deadly combination, proven in our cohort, adding on to the current evidence in the literature. We noted that our STEMI CKD patients tend to be younger than the Caucasian with extremely high prevalence of diabetes mellitus. The poor outcome mainly driven by immediate or short term adverse events peri-procedural, therefore suggesting that more efficient treatment in this special group is imperative.

## Background

Cardiovascular disease remained the most common cause of death in patients with non – dialysis dependent chronic kidney disease (CKD) or end-stage renal disease (ESRD) alike [1–4]. Pre-existing renal impairment or as a consequence of myocardial infarction are both associated with poor clinical outcome [5]. In fact, presence of any forms of renal insufficiency in ST elevation myocardial infarction (STEMI) patients is associated with higher cardiovascular mortality and morbidity [6, 7]. Patients with CKD are often underrepresented in clinical trials resulting in lack of evidence concerning the best mode of STEMI treatment in this subgroup [8]. However, among STEMI survivors, patients with CKD do not necessarily have poorer health status as compared to their non-CKD counterparts [9].

Modes of revascularization in STEMI patients with CKD have always been a dilemma among cardiologist. CKD patients with STEMI tend to receive lower rates of evidence-based therapies [10, 11]. In the setting of STEMI, primary percutaneous coronary intervention (PCI) is the cornerstone of treatment regardless the status of patient’s renal functions [12, 13]. The poor outcome of CKD following acute myocardial infarction may be related to them having more severe coronary lesions or to the higher burden of pre-morbid conditions often associated with CKD. Also, PCI is in itself an invasive procedure with risks involved. CKD patients have higher tendency to develop PCI related complications both locally and systemically. The risk of major complications of PCI such as contrast-induced nephropathy (CIN) and bleeding probably contributes further to the poor outcome. Therefore, the administration of invasive coronary revascularization and evidence-based pharmacotherapy may paradoxically have deleterious effect if not done with great care and timely manner.

For these reasons, there bound to be a spectrum of disparity and inconsistency in terms of hospital management and hence clinical outcome of these patients. Thus, this study focuses on STEMI patients with renal impairment treated with PCI by means of the Malaysian National Cardiovascular Disease Database-Percutaneous Coronary Intervention (NCVD-PCI) registry involving 15 hospitals across the nation. We aim to assess the clinical characteristics, procedural details, mortality and other major cardiovascular events associated with this sub-set of patients.

## Methods

### Study population

The NCVD-PCI registry is an on-going collaboration between the Ministry of Health Malaysia and the National Heart Association of Malaysia. The data of patients underwent PCI from 2007 to 2014 in 15 participating hospitals (13 public and 2 private) across Malaysia was captured using standardized case report forms. The list of participating hospitals can be found in the Annual Report of the NCVD-PCI registry year 2013–2014 [14]. A unique national identification number was assigned to each patient to avoid duplication and maintain anonymity. Patient’s baseline characteristics, risk factor profile, extent of coronary artery disease, revascularization methods and estimated glomerular filtration rate (eGFR) were recorded. Follow-up was done at 1 year after hospital discharge via phone call or when the patient came to the clinic for review. Verified data will be entered using an established electronic data acquisition form with built-in plausibility checks [14].

### Definitions

The patients were categorized into two groups; CKD and non-CKD. CKD is defined as GFR of < 60 mls/min/1.73m^2^ as determined by Modification of Diet in Renal Disease (MDRD) formula [15–17]. In this registry, CKD and ESRD were combined as a single group. Therefore, we are unable to perform separate analysis for both conditions. STEMI was defined as persistent ST segment elevation ≥1 mm in two contiguous electrocardiographic leads, or the presence of a new left bundle branch block in the setting of positive cardiac biomarkers.

Data from this registry depends heavily on patients self-reporting for baseline characteristics and co-morbidities (self-recall, previous hospital’s discharge letter or list of medications). Apart from that, the information was cross-checked with patient’s medical records, laboratory results and pre-procedural notes. Malaysia is a unique multi-ethnic country with diversified races and religions. The 3 major ethnic groups are Malay, Chinese and Indian. The rest of the study population (approximately 5%) is categorized into others; these include other native people, non-native Malaysian, foreigner and unknown status. Single vessel disease is defined as lesions > 50% stenosis in only 1 major epicardial vessel, whereas multi-vessels disease is defined as lesions > 50% in 2 or more epicardial vessels. Lesion type is divided according to the American Heart Association / American College of Cardiology (AHA/ACC) classification [18]. Since this registry only enrolled patients who underwent PCI, data on thrombolytic therapy for STEMI was not captured. However, patient who underwent rescue PCI may represent most of the patients who might have received thrombolytic therapy as first-line treatment. The procedural complications were defined as per the NCVD data definition form document [14]. For the pharmacological treatment, only information from the hospital’s discharge document is recorded.

### Statistical analysis

The study populations were STEMI patients stratified by presence or absence of CKD. Continuous variables were described as mean (SD) if normally distributed and compared using the Student’s *t-*test or as median (interquartile range) if skewed and compared using the Mann-Whitney *U* test. Categorical variables were described as numbers (percentages). Comparisons of categorical data were analyzed using the chi-square test or Fisher’s exact test. To evaluate the association between CKD and mortality within 1 year, their respective multivariable-adjusted hazard ratios (HR) were calculated using Cox proportional-hazards regression models. Variables included in the model were chosen by separate univariate analyses; those with *p*-value of < 0.05 were included in the final model. The variables were entered stepwise into the model using the forward likelihood ratio method with p-in: 0.05 and p-out: 0.10. Multicollinearity between the included variables was examined using standard error of b coefficient. All tests were two sided and a *p*-value of less than 0.05 was considered to be statistically significant. The assumption of proportional hazards for each covariate was reviewed separately by the means of log-minus-log survival plots. Hazard ratios were reported together with the 95% confidence interval (CI) values. All statistical analyses were performed using SPSS version 23. Missing data were assumed to be missing completely at random (MCAR) based on the Little’s MCAR test p-value of more than 0.05. These missing data were omitted automatically from the analysis by list-wise deletion.

### Ethic statement

The NCVD-PCI is registered in the National Medical Research Register of Malaysia (ID: NMRR-07-20-250) and received ethical approval from the Ministry of Health Medical Research and Ethics committee. Consent from individual patients was not necessary as the data collected were anonymized. Each patient will receive a unique identification number recorded in the registry.

## Results

### Baseline characteristics

A total of 6563 patients (almost 80% of total number of STEMI patients treated with PCI during study period) were included in the final analysis, 5765 (87.8%) men and 798 (12.2%) women. Patients with CKD were numerically older than their counterpart without CKD. Ethnic distribution depicted general Malaysian population with Malay being the most dominant ethnic group. In terms of co-morbidities, patients with CKD tend to have more conventional cardiovascular risk factors except for cigarette smoke exposure. Baseline characteristics of study population further elaborated in Table 1.

**Table 1:** Baseline characteristics

Characteristics	CKD *N* = 1516	Non-CKD *N* = 5047	*p*-value
Age (year)	61.02 ± 9.95	53.75 ± 10.24	0.539
Gender			
Male	1213 (80.0)	4552 (90.2)	< 0.001
Female	303 (20.0)	495 (9.8)	
Ethnicity			
Malay	937 (61.8)	2709 (53.8)	< 0.001
Chinese	259 (17.1)	954 (18.9)	
Indian	205 (13.5)	1009 (20.0)	
Others	115 (7.6)	368 (7.3)	
BMI (kg/m^2^)	26.55 ± 4.59	26.32 ± 4.39	0.244
Smoking status			
Current smoker	370 (29.3)	2083 (45.4)	< 0.001
Never/former smoker	892 (70.7)	2506 (54.6)	
Medical history			
Diabetes mellitus	777 (54.6)	1869 (39.4)	< 0.001
Hypertension	1145 (79.2)	2836 (59.8)	< 0.001
Dyslipidemia	938(68.8)	3020 (66.2)	0.073
Cerebrovascular disease	47 (3.2)	67 (1.4)	< 0.001
Coronary artery disease	634 (43.9)	2037 (41.9)	0.182
Heart failure	75 (5.1)	141 (2.9)	< 0.001

All values are n, (%) unless stated. Percentages for variables under the medical history category are calculated from a total that includes the unknown category

### Angiographic characteristics

STEMI patients with CKD have more extensive coronary artery disease with higher rate of multi-vessels disease and more complex coronary lesions (type B2 / C and left main-stem involvement). The use of drug eluting stents (DES) which is regarded as the benchmark in PCI is lower in CKD (Table 2).

**Table 2:** Lesion characteristics and procedural data

Variable	No. (%)		*p*-value
	CKD	Non-CKD	
Number of lesions	1655 (23.4)	5423 (76.6)	
Single vessel	460 (45.7)	1826 (56.5)	< 0.001
Multi-vessels	546 (54.3)	1408 (43.5)	
AHA/ACC type			
A & B1	523 (32.3)	2061 (38.9)	< 0.001
B2/C	1097 (67.7)	3242 (61.1)	
Chronic total occlusion	93 (5.6)	276 (5.1)	0.396
Vessels involved			
LMS	45 (2.7)	59 (1.1)	< 0.001
LAD	695 (42.0)	2552 (47.1)	< 0.001
RCA	488 (29.5)	1384 (25.5)	0.001
LCX	146 (8.8)	477 (8.8)	0.974
Stent Type			
BMS	578 (38.8)	1464 (29.5)	< 0.001
DES	811 (54.4)	3070 (61.8)	
Others	102 (6.8%)	437 (8.8)	

All values are n, (%) unless stated

### Modes of treatment for STEMI

There was notably higher percentage of CKD patients with STEMI present in severe acute left ventricular dysfunction (Killip class 3 and 4; 24.9% in CKD vs 8.7% in non-CKD). Higher percentages of rescue PCI in both arms suggested that thrombolytic therapy was the default mode of treatment of STEMI in this population. Primary PCI, which is the preferred revascularization strategy was only performed in 20.7% among CKD patients and 15.5% among the non-CKD patients (Table 3).

**Table 3:** Modes of treatment for STEMI

Variable		CKD	Non-CKD	*p*-value
Killip Class	Class 1 & 2	925 (75.1)	3793 (91.3)	< 0.001
	Class 3 & 4	306 (24.9)	362 (8.7)	
PCI Status	Rescue	498 (69.6)	1501 (71.1)	0.002
	Primary	148 (20.7)	326 (15.5)	
	Facilitated	5 (0.7)	20 (0.9)	
	Delayed	65 (9.1)	263 (12.5)	

All values are n, (%) unless stated

### Medications on discharge

Table 4 showed the medications prescribed at discharge for this study cohort. Usage of anti-platelets therapy was almost similar between the 2 groups, although the percentage of patients with CKD who were prescribed aspirin was lower. Combination of aspirin and clopidogrel remained the most commonly prescribed dual anti-platelet regime. It is obvious that the use of more potent newer generation of anti-platelets such as ticagrelor and prasugrel was low, below 10% of the population. Although CKD patients had more co-morbidity, they were given less of evidence-based medications. Except beta-blocker, the use of statin, angiotensin converting enzyme inhibitor (ACE-I) and angiotensin receptor blocker (ARB) was consistently lower in CKD patients in comparison to their non-CKD counterparts.

**Table 4:** Medications on discharge

Medication on discharge, n (%)	No. (%)		*p*-value
	CKD	Non-CKD	
Aspirin	1219 (95.2)	4617 (96.7)	0.011
Clopidogrel	1163 (90.9)	4326 (90.7)	0.826
Statin	1184 (92.8)	4514 (95.2)	0.001
Beta-blocker	957 (75.9)	3549 (75.7)	0.881
ACE-I/ARB	745 (59.6)	3198 (68.3)	< 0.001

All values are n, (%) unless stated

### Procedural complications

Patients with CKD are more likely to develop procedural related complications (Table 5). Vascular complications include bleeding, access site occlusion, loss of distal pulse, dissection and pseudoaneurysm. Major adverse cardiovascular events (MACE) included periprocedural MI, emergency PCI, bailout CABG, cardiogenic shock, arrhythmia, transient ischemic attack/stroke, cardiac tamponade and heart failure. Death was analyzed as a separate outcome.

**Table 5:** In hospital procedural complications

Complications, n (%)	No. (%)		*p*-value*
	CKD	Non-CKD	
Vascular complications**	20 (1.3)	27 (0.5)	0.001
MACE***	107 (7.1)	130 (2.6)	< 0.001
Death	198 (13.2)	120 (2.4)	< 0.001

All values are n, (%) unless stated

*Chi square test, Pearson’s *p*-value

**Vascular complications included bleeding, access site occlusion, loss of distal pulse, dissection, pseudoaneurysm

***MACE (major adverse cardiovascular events) included periprocedural MI, emergency PCI, bailout CABG, cardiogenic shock, arrhythmia, TIA/stroke, cardiac tamponade and heart failure

### Outcome

**Fig. 1:**
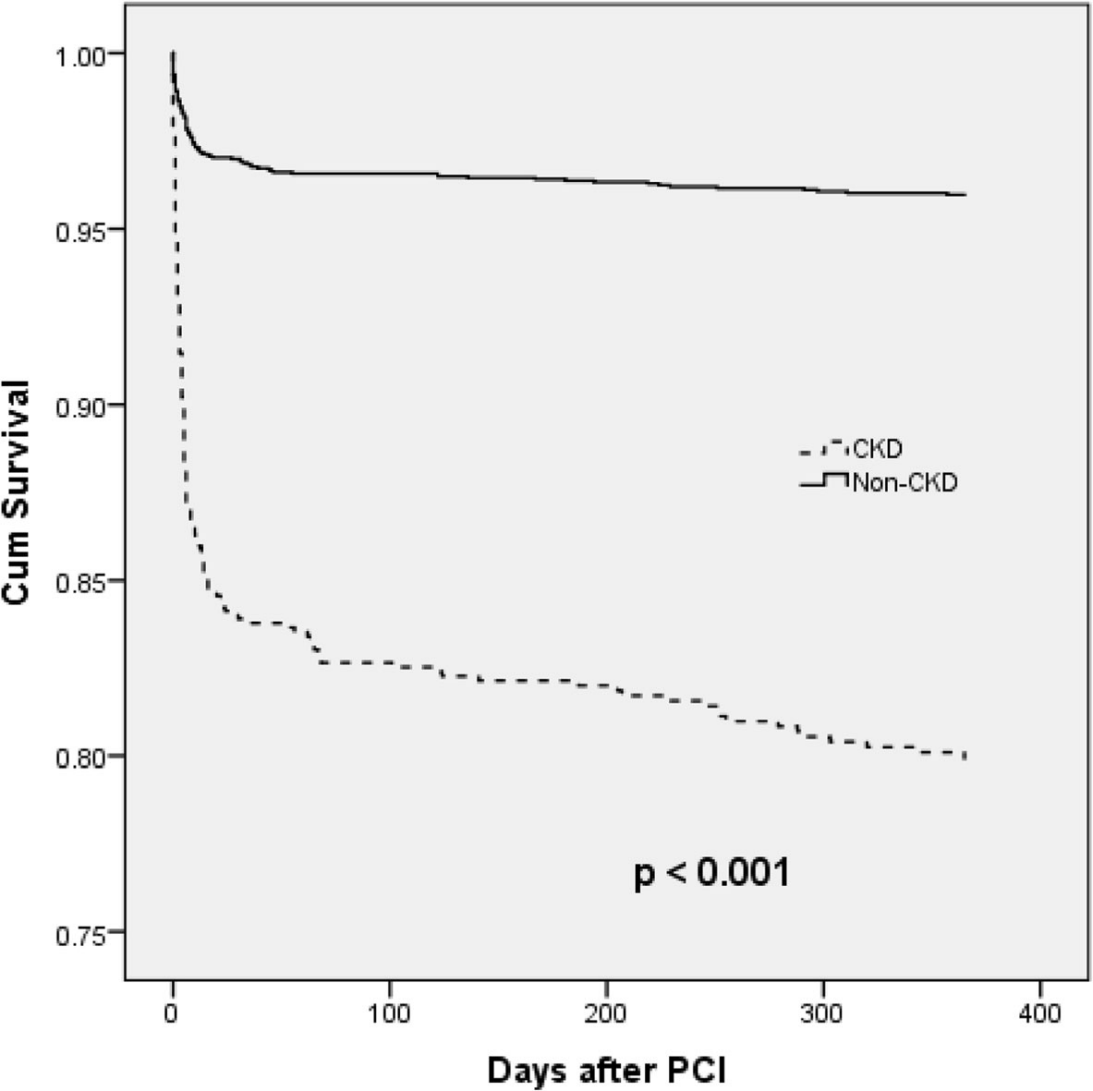
Kaplan Meier curve showing the cumulative survivals between those with and without CKD up to 1 year after the index PCI

Table 6 shows the odd ratios of developing in-hospital vascular complications, MACE and death in patients with CKD. After adjustment of the covariates, patients with CKD were more likely to develops in-hospital death (OR: 4.5, 95% CI 3.11–6.65) and in-hospital MACE (OR: 3.42, 95% CI 2.39–4.90) compared to non-CKD patients. Figure 1 showed the Kaplan Meier survival curves between CKD and non-CKD patients up to 1 year of follow up, with the CKD group had significantly lower cumulative survival after PCI compared to the non-CKD group. The early phase mortality contributes the most in the difference. Further analysis using multivariate Cox proportional hazard regression was done to adjust for the significant covariates, and it can be seen that patients with CKD had significantly higher risk of 1 year mortality (HR: 3.79, 95% CI 2.84–5.07) compared to the non-CKD group (Table 7).

**Table 6:** Odd ratios for in-hospital vascular complications, MACE and death for patients with GFR < 60 mls/min/1.73m^2^ using multivariate logistic regression

Outcome	Unadjusted OR	*p*-value and 95% Confidence Interval	Adjusted OR^a^	*p*-value and 95% Confidence Interval
Vascular Complication	2.49	0.002(1.39–4.45)	1.88^b^	0.07 (0.95–3.7)
In Hospital MACE	2.88	< 0.001(2.21–3.74)	3.42^c^	< 0.001(2.39 – 4.90)
In Hospital Death	6.17	< 0.001 (4.88–7.81)	4.55^d^	< 0.001 (3.11–6.65)

^a^Only those variables with *p* value < 0.2 from separate univariate analyses were included in the final model to calculate the odds ratio

^b^adjusted for gender, hypertension and Killip class

^c^adjusted for gender, race, smoking status, diabetes mellitus, dyslipidemia, heart failure, previous PCI, Killip class and age > 60 years

^d^adjusted for gender, race, smoking status, diabetes mellitus, dyslipidemia, hypertension, heart failure, history of cerebrovascular accident, Killip class and age > 60 years

**Table 7:** Hazard ratios for 1 year mortality for patients with GFR < 60 mls/min/1.73m^2^ using Cox Proportional Hazard Regression

	Unadjusted HR	*p*- value and 95% Confidence Interval	Adjusted HR^a^	*p*- value and 95% Confidence Interval
1-year mortality	5.44	< 0.001 (4.41–6.71)	3.79	< 0.01 (2.84–5.07)

^a^Adjusted for gender, race group, dyslipidemia, diabetes mellitus, hypertension, heart failure, history of cerebrovascular accident, Killip class and age > 60 years

## Discussions

CKD and STEMI is a deadly combination that is not so uncommonly encountered. National Cardiovascular Data Registry-Acute Coronary Treatment and Intervention Outcomes Network (NCDR-ACTION) reported prevalence of 30.5% among patients presenting with STEMI and 42.9% among patients presenting with non-ST segment elevation myocardial infarction (NSTEMI) in the United States [10]. Acute coronary syndrome (ACS) in patients with CKD has been associated with higher rates of mortality and bleeding [19–21]. This special group of patients is less likely to receive evidence-based therapy and often left out from randomized controlled trials. Hence, data from real-world registries like ours could contribute to the knowledge on how to best manage these high-risk patients.

The prevalence of CKD among our STEMI patients is 23.1% (only include patients with available eGFR data). The high number most likely contributed by the large percentage of diabetics in our country at 17.5% [22]. According to the Malaysian Dialysis and Transplant Registry, 61% of new dialysis patients in 2014 had diabetes as the cause of primary renal disease [23]. Diabetics also known to present with more diffuse and complex coronary lesions. This by itself could lead to adverse outcomes among STEMI patients. In our cohort of STEMI with CKD, more than half were diabetics. This number is significantly higher than reported in SWEDEHEART registry, which have diabetes rate between 25.8% in men and 28.2% in women [24]. Glycemic control optimization especially in those patients with diabetic nephropathy is important, since CKD and cardiovascular diseases seem to have synergistic effects. Cardiovascular disease has consistently contributed to more than 30% of mortality among patients with CKD and ESRD in Malaysia over a decade [23]. More stern action therefore has to be taken by the lawmakers to improve this alarming situation.

As documented before, our STEMI patients tend to be much younger than the Caucasian [25]. In this particular cohort as well, although CKD with STEMI patients were numerically older than their non-CKD counterpart, they were significantly younger than the CKD cohort of GRACE registry by more than 10 years [26]. The findings suggest that screening for cardiovascular disease and CKD should start much earlier in our population in order to be able to prevent CKD related cardiovascular outcomes and vice versa.

PCI facilities are not widely available throughout Malaysia. Although primary angioplasty is the recommended treatment for STEMI in Malaysia, in line with major international guidelines, we are still held up by the number of PCI capable centers in the public sector. Thrombolytic therapy remained an important mode of revascularization in patients presenting with STEMI in most hospitals. In this cohort, almost 70% received PCI as rescue procedure after failure to response to thrombolytic therapy. The need for rescue PCI signifies higher risk of bleeding and adverse events. The less use of DES in STEMI CKD patients could also contribute to the poorer outcome. However, this has to be determined in future sub-analysis study.

Not only that, patients with CKD did receive less of evidence-based treatments upon discharge from the hospital after an episode of STEMI. Prescription for aspirin was less in CKD patients most likely because they are generally deemed ‘high bleeding’ risk group, which could be predisposed by uraemic gastropathy, although not entirely true [27]. In terms of statin, there are conflicting evidences exist whether statin therapy would change the progression of chronic kidney disease [28, 29]. Prescribing statin solely for renal protective effects is currently not recommended. However, statin in high cardiovascular risk patients’ evidence is overwhelming [30]. It is also interesting to note that the use of renal angiotensin system blocker was lower in the CKD patients despite the general recommendation for this particular group of drugs in CKD patients [31–33]. We assume that this could be due to prescriber bias, worry of increasing serum creatinine level as well as hyperkalaemia.

Treatment and management in the early phase of STEMI is crucial in CKD patients. According to the Kaplan-Meier curve, the difference in outcome occurs earlier rather than later. This suggests that CKD patients do not tolerate the insult of STEMI and consequence PCI as well as the non-CKD patients. They were more likely to develop in-hospital complications peri-procedural and significantly more patients died during the same admission. The trends continued even after they are discharged. At 1 year after the index PCI, CKD patients with STEMI were 3.79 times more likely to die as compare to non-CKD patients. For future improvement, the treatment and monitoring of CKD in STEMI / PCI should be intensified in the early phase. Modifiable prognostic indicators have to be optimized in CKD patients.

As this is a registry-based study, there are limitations worth to note. First, this is a retrospective study of the data collected from a nation-wide registry. Various factors could contribute to the compliance of the data entry by respective sites. Missing data is the most important issue that needs to be dealt with using statistical analysis. We have opted to the list-wise deletion technique in dealing with missing data rather than the much-preferred multiple imputation technique, hence leading to possibility of unmeasured or residual confounding. Apart from that, the presence of missing values in the outcome data may lead to information bias.

Second, we did not divide further the different stages of CKD as the number in each sub-group deemed to be too small for meaningful analysis. However, analyzing them as just 2 major sub-groups could potentially introduce bias. For example, patients with ESRD may behave differently from patients in CKD stage 5. Unfortunately, the information on dialysis is lacking that we need to drop it out from the analysis.

Third, the estimated GFR formula adopted in this registry is MDRD. We know now that there is growing evidence to suggest that MDRD may not be as accurate as newer GFR estimates formula such as Chronic Kidney Disease Epidemiology Collaboration (CKD-EPI). However, the comparison between these 2 GFR estimates formula has never been validated specifically in our multi-ethnic population. The serial readings of serum creatinine post PCI were also not available for interpretation. We will not be able to see presence of CIN in CKD patients who underwent PCI in this cohort.

Finally, PCI techniques may have undergone a significant change within the eight years of this registry data. The way that patients were treated, and their outcomes could have been different. There might also be inter-hospital variations that we are not able to take into account for when determining the outcomes. It is likely that patients treated at PCI capable hospitals expected to fare better as their counterpart treated at non-PCI capable hospitals.

## Conclusion

We conclude that CKD patients made up a significant proportion of all PCI-treated STEMIs (23.1%). Hence, they are an important non-negligible group of high-risk patients. CKD patients are associated with many other unfavourable baseline characteristics as well as more severe coronary lesions. Due to the above, the outcome is poorer as expected. The difference in outcome is most obvious at early stage post STEMI and PCI. Hence, we urge the parties involved to improve awareness among at risk population and implementation of more efficient prompt treatment in this special sub-set of patients.

## Abbreviations

ACC: American College of Cardiology
ACE-I: Angiotensin converting enzyme inhibitor
ACS: Acute coronary syndrome
AHA: American Heart Association
ARB: Angiotensin receptor blocker
BMS: Bare metal stent
CIN: Contrast-induced nephropathy
CKD: Chronic kidney disease
CKD-EPI: Chronic Kidney Disease Epidemiology Collaboration
DES: Drug-eluting stent
eGFR: Estimated glomerular filtration rate
ESRD: End-stage renal disease
GFR: Glomerular filtration rate
LAD: Left anterior descending artery
LCx: Left circumflex artery
LMS: Left main stem artery
MACE: Major adverse cardiovascular events
MDRD: Modification of Diet in Renal Disease
NCVD-PCI: National Cardiovascular Disease Database-Percutaneous Coronary Intervention
NSTEMI: Non ST-elevation myocardial infarction
PCI: Percutaneous coronary intervention
RCA: Right coronary artery
STEMI: ST-elevation myocardial infarction
